# MDM2 regulates the stability of AR, AR-V7, and TM4SF3 proteins in prostate cancer

**DOI:** 10.1530/EO-23-0017

**Published:** 2024-02-09

**Authors:** Prabesh Khatiwada, Ujjwal Rimal, Zhengyang Han, Lirim Shemshedini

**Affiliations:** 1 Department of Biological Sciences, University of Toledo, Toledo, Ohio, USA; 2 Center for Translational Immunology, Columbia University, New York, New York, USA; 3 Dana-Farber Cancer Institute, Harvard University, Boston, Massachusetts, USA

**Keywords:** AR, AR-V7, TM4SF3, MDM2, prostate cancer

## Abstract

Androgen receptor (AR) and its constitutively active splice variant, AR Variant 7 (AR-V7), regulate genes essential for the development and progression of prostate cancer. Degradation of AR and AR-V7 by the ubiquitination proteasomal pathway is important for the regulation of both their protein stability. Our published results demonstrate that the interaction of TM4SF3 with either AR or AR-V7 leads to mutual stabilization due to a reduction in their ubiquitination and proteasomal degradation. These results led us to search for a common E3 ligase for AR, AR-V7, and TM4SF3. Depletion by siRNA of several E3 ligases identified MDM2 as the common E3 ligase. MDM2 inhibition by siRNA depletion or using a pharmacological inhibitor (MDM2i) of its E3 ligase activity led to elevated levels of endogenous AR, AR-V7, and TM4SF3 in prostate cancer cells. MDM2 knockdown in PC-3 cells, which do not express AR, also increased TM4SF3, demonstrating that MDM2 affects the TM4SF3 protein independent of AR. We further demonstrate that MDM2i treatment reduced the ubiquitination of AR and TM4SF3, suggesting that MDM2 can induce the ubiquitination of these proteins. Increased AR and AR-V7 protein levels induced by MDM2i treatment resulted in the expected increased expression of AR-regulated genes and enhanced proliferation and migration of both LNCaP and Enzalutamide-resistant CWR-22Rv1 prostate cancer cells. Thus, our study expands the known roles of MDM2 in prostate cancer to include its potential involvement in the important mutual stabilization that TM4SF3 exhibits when interacting with either AR or AR-V7.

## Introduction

Androgen receptor (AR) is a transcription factor critical for prostate cancer development and progression (Heinlein & Chang 2004). AR is normally sequestered and inactivated by binding to heat shock protein 90 (HSP90) in the cytoplasm, which assists in AR folding and maturation (Pratt & Toft 1997, Buchner 1999, Georget *et al.* 2002). After binding to androgen, AR undergoes conformational changes, dissociates from HSP90, dimerizes, and shuttles into the nucleus, where it binds to the androgen-responsive elements (AREs) of androgen-regulated genes (Huang & Tindall 2002, Dehm & Tindall 2006). Prostate cancer cell proliferation and survival are driven by AR, and tumors initially respond to androgen-deprivation therapy (ADT). However, the disease inevitably relapses into a more aggressive androgen-refractory stage, referred to as castration-resistant prostate cancer (CRPC) (Taplin & Balk 2004, Hendriksen *et al.* 2006). AR signaling remains critical for the survival of CRPC, and tumors use different mechanisms to bypass the need for androgen for AR activation. One of these mechanisms may be the expression of AR-V7, a constitutively active splice variant of AR that lacks the ligand-binding domain (LBD) (Antonarakis *et al.* 2014, Sharp *et al.* 2019). AR-V7 is known to be associated with HSP70 in the cytoplasm (Liu *et al.* 2018). AR and AR-V7 transcriptionally regulate a number of genes that are essential for prostate cancer cell survival (Lu *et al.* 2015, Shafi *et al.* 2015). Both AR and AR-V7 are known to have several posttranslational modifications that regulate their stability, turnover, and transcriptional output (Gioeli & Paschal 2012, van der Steen *et al.* 2013).

Ubiquitination is a reversible covalent process that results in the attachment of ubiquitin to specific substrates by the sequential action of enzymes E1, E2, and E3 (Schlesinger *et al.* 1975). Ubiquitin molecules are commonly added to specific lysine residues in the substrate by the specific action of an E3 ligase, which also determines the kind of ubiquitination will be added (Hershko & Ciechanover 1998, Sommer & Wolf 2014). Ubiquitination of proteins can be mono- or different kinds of poly-ubiquitination (Swatek & Komander 2016). Mono-ubiquitination is generally associated with DNA repair and transcriptional regulation, while K48 poly-ubiquitination is associated with proteasomal degradation, and K63 poly-ubiquitination with cellular signaling (Komander 2009, Swatek & Komander 2016). AR is well known to be degraded by ubiquitin proteasome system (UPS), and its ubiquitination sites as well as several E3 ligases have been identified (Li *et al.* 2014). E3 ligases that have been reported to act on AR include MDM2, STUB1, RNF6, and CHIP (Li *et al.* 2014). On the other hand, UPS degradation of AR-V7 is less explored, with studies showing that STUB1 (Liu *et al.* 2018) and MDM2 (Li *et al.* 2015) can promote the proteasomal degradation of AR-V7.

Proteasomal degradation of TM4SF3, in contrast to AR and AR-V7, was unknown until our two recent studies, showing that MG132 can block TM4SF3 degradation in prostate cancer cells (Bhansali *et al.* 2016), and demonstrated TM4SF3 *in vitro* ubiquitination that is inhibited by association with either AR or AR-V7 (Khatiwada *et al.* 2023). In view of these findings, we set up a screen of E3 ligases known to act on AR ubiquitination to identify a common E3 ligase for all three proteins. This led us to MDM2, a RING domain-containing E3 ligase well characterized for its role in the p53 ubiquitin-proteasomal degradation pathway (Michael & Oren 2003, Moll & Petrenko 2003). MDM2 was also the first E3 ligase shown to promote AR ubiquitination (Giridhar *et al.* 2019), and has recently been identified as an E3 ligase that induces AR K311 ubiquitination (McClurg *et al.* 2017). In this study, we found that inhibiting MDM2, either by siRNA or a pharmacological inhibitor of its RING domain, increased levels of AR, AR-V7, and TM4SF3 protein in prostate cancer cells, leading to elevated growth of these cells.

## Materials and methods

### Cell culture and treatments

LNCaP, CWR-22RV1, and PC-3 cells were purchased from ATCC and maintained between passage 9 and 35, in RPMI-1640 (Corning) medium supplemented with 10% fetal bovine serum (FBS) (Cytiva, Marlborough, MA, USA) and penicillin–streptomycin (Corning). R49F cells were kindly gifted to us by Dr Anima Zoubedi and maintained in the presence of 10 μM enzalutamide (Cayman Chemical) in RPMI medium, similar to other prostrate cancer cells. HEK-293 cells were maintained in in DMEM (Corning) medium supplemented with 10% FBS and penicillin–streptomycin.

To maintain basal levels of AR/AR-V7 and TM4SF3, cells were grown in RPMI-1640 or DMEM medium (HEK-293T) without phenol red and l-glutamine, supplemented with 2% dextran charcoal extracted FBS (2% DCC) (Gibco), also referred as deprivation medium, for 48 h before MDM2 inhibitor (MDM2i) treatment or transfections. For androgen treatments, cells were grown in deprivation medium for 48 h, before treatment with ethanol or 10 nM R1881 (Sigma-Aldrich).

### Plasmids and transfection

The MDM2 expression plasmid was kindly gifted to us by Dr Kam Yeung. We PCR-amplified pEGFF-C1-AR (Addgene, Watertown, MA, USA), pEGFP-C1-AR-V7 (Addgene), and pCMV6-TM4SF3-Myc-DDK (OriGene, Rockville, MD, USA) plasmids using appropriate primers (Supplementary Table 1, see section on supplementary materials given at the end of this article) and cloned into MluI/BamH1-digested pLenti6.3/AR-GC (Addgene) plasmid using the NEBuilder HighFi DNA assembly kit (NEB). The pRK5-HA-ubiquitin (Addgene) was purchased from Addgene. The plasmids were transfected into HEK-293T cells grown in deprivation medium for 48 h before transfection using polyethyleneimine (PEI) (PolyScience, Niles, IL, USA) reagent, following the manufacturer’s recommendation.

### siRNA and MDM2 inhibition

siRNAs against MDM2, CHIP, STUB1, or RNF6 were transfected into prostate cancer cells grown in deprivation medium for 48 h using GenJet In Vitro siRNA Transfection Reagent (SignaGen, Frederick, MD, USA) at a 50 nM concentration, following the manufacturer’s instructions. After 24 h of transfection, we changed the medium and allowed the cells to grow for an additional 48 h before lysing the cells for protein or mRNA extraction.

We inhibited MDM2 E3 ligase activity in prostate cancer cells by treating cells grown in deprivation medium for 48 h with the indicated concentrations of MDM2 E3 Ligase Inhibitor II HLI373 (MDM2i) (Sigma-Aldrich) (Giridhar *et al.* 2019). Following treatment, the cells were grown for another 48 h before being lysed for protein or mRNA extraction.

### Cell proliferation and migration

To study the effect of MDM2i on prostate cancer cell proliferation, LNCaP, CWR-22RV1, and PC-3 cells were plated in 96-well plates in full serum or grown in deprivation medium for 48 h before treatment with vehicle or the indicated concentration of MDM2i. Cell proliferation was determined by the MTT assay, as described previously (Bhansali *et al.* 2016).

Cell migration was measured using the Cytoselect 24-Well Cell Migration Assay kit, 8 µm (fluorometric quantitation) (Cell Biolabs, San Diego, CA, USA) following the manufacturer’s protocol. Briefly, a cell suspension containing 100,000 cells/mL (in deprivation medium treated with vehicle or MDM2i) was used to monitor cell migration into the lower chamber of the cell migration assay kit, which contained RPMI-1640 medium with 10% DCC-stripped serum. After 48 h of incubation at 37^°^C, cells were quantified by fluorescence using a microplate reader. The cell migration values (folds) are normalized to control, and they represent the average of three independent experiments plus s.d..

### Western blotting and immunoprecipitation

Western blotting and immunoprecipitation (IP) were performed as described previously. Cells were extracted using M-Per Mammalian Protein Extraction Reagent (Thermo Fisher), protein concentration was measured using Bradford, and equal amounts of whole cell lysates were loaded onto the gel before Western blotting using antibodies against AR, AR-V7, MDM2, HA, and Myc (all from Cell Signaling Technology), TM4SF3 and β-actin (both from Invitrogen), and FLAG (Sigma-Aldrich). For endogenous IP, antibodies against AR, AR-Carboxy terminal (Cell Signaling Technology), AR-V7, MDM2, and TM4SF3 were used to pull down respective proteins. For exogenous IP, FLAG-AR, FLAG-AR-V7, TM4SF3-Myc-DDK, and MDM2 were expressed and antibodies against the FLAG epitope, Myc epitope, or MDM2 were used.

### Ubiquitination

HEK-293T cells grown in deprivation medium were transfected with appropriated plasmids using PEI (PolyScience) in 60 mm dish. The transfected cells were treated with ethanol or 5 μM MDM2i for an additional 42 h, before treatment with 20 μΜ ΜG132 for another 6 h before cell lysis. Cell lysis, denaturation, and IP were performed using the protocol described in Choo and Zhang (2009). Ubiquitination was detected following an anti-Flag IP and Western blotting using an anti-HA antibody.

### Real-time PCR

LNCaP, CWR-22RV1, and PC-3 cells were grown in full serum or deprivation medium for 48 h before siRNA transfection or treatment with MDM2i, as indicated in the figures. After 48 h of transfection or treatment, RNA isolation was performed using the TRIZOL Reagent (Invitrogen) following the manufacturer’s instructions, and quantitative real tme-PCR (qRT-PCR) was conducted using iQ SYBR Green Supermix (Bio-Rad). The results were quantified following the 2^−ΔΔCt^ method (Livak method) and are given relative to GAPDH expression, representing the average of three replicates plus s.d. The PCR primers were purchased from IDT Technologies, and the upstream and downstream primers, respectively, used for each gene were: GAPDH, 5′-CGAC CACTTTGTCAAGCTCA-3′ and 5′-AGGGGAGATTCAGTGTGGTG-3′; AR, 5′-GCATGGCAGAGTGCCCTATC-3′ and 5′-TCCCAGAGTCATCCCTGCTTCAT-3′; AR-V7, 5′-AAGAGCCGCTGAAGGGAAAC-3′ and 5′-TGCCAACCCGGAATTTTTCTC-3′; TM4SF3, 5′-GGCTTCCTGGGATGCTGCGG-3′ and 5′-GTCGCCACCTGCAGGAGCAG-3′; PSA, 5′-GCAGCATTGAACCAGAGGAG-3′ and 5′-CCCATGACGTGATACCTTGA-3′; TMPRSS2, 5′-CCTCTAACTGGTGTGATGGCGT-3′ and 5′-TGCCAGGACTTCCTCTGAGATG-3′; IGF1, 5′-CAACATCTCCCATCTCTCTG-3′ and 5′-GAAATCACAAAAGCAGCACT-3′; ADAM9A, 5′-GAATGCACAAGAACCACAAT-3′ and 5′-TAGGAAGCTACTAGGAGACA-3′; RAP2A, 5′-GATTCAGAGGCCTTCTAGTG-3′, and E2F7, 5′-TGTATCTTTAAGGAAGCCCT-3′ and 5′-CGTCGACGTTCAACATTAAG-3′.

## Results

### MDM2 downregulates the AR and TM4SF3 protein levels by inducing their ubiquitination

**Figure 1:**
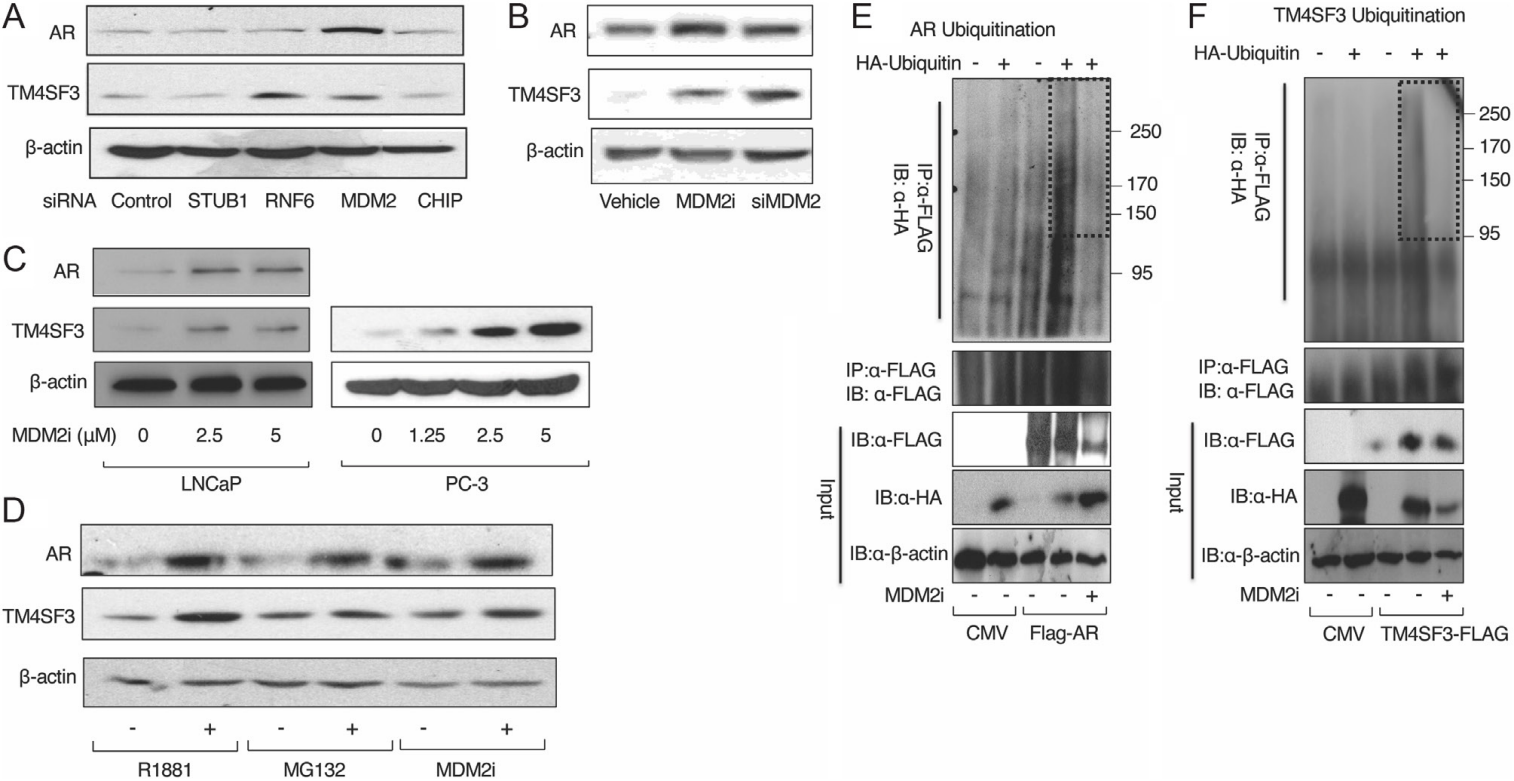
MDM2 regulates AR and TM4SF3 protein levels. LNCaP cells were grown in 2% DCC for 48 h before (A) transfection with indicated siRNAs, (B) treatment with MDM2i, or transfection with siMDM2, (C) treatment with vehicle (0) or 2.5 and 5 μM MDM2i, or (D) treatment with vehicle (−), 10 μM MG132 or 5 μM MDM2i. After 48 h, or transfection or treatment, cells were lysed with M-PER, and Western blotting was performed to measure AR and TM4SF3 protein levels. (E, F) HEK-293 cells grown in 2% DCC were co-transfected with FLAG-AR (E) or TM4SF3-FLAG (F) and either empty or HA-Ubiquitin and treated with either vehicle (−) or 5 μM MDM2i for 24 h. After 24 h, cells were treated with 10 μM MG132 for another 6 h before performing a denatured cell lysis and IP with anti-FLAG antibody. Following the IP and Western blot, ubiquitination was detected by blotting the proteins with anti-HA antibodies. AR or TM4SF3, and HA-Ubiquitin were detected in input by using indicated antibodies. In all Western blots, β-actin was used as a loading control and is shown here as a representative of three independent experiments. Note that the vehicle is DMSO.

Since the E3 ligases MDM2, RNF6, CHIP, and STUB1 have been shown previously to act on AR (Li *et al.* 2014), we decided to use the siRNA knockdown approach (Supplementary Fig. 1) to examine each for co-regulation of AR and TM4SF3 in LNCaP cells. Interestingly, knockdown of MDM2 (Supplementary Fig. 2) resulted in increased levels of both AR and TM4SF3, while RNF6 knockdown increased levels of only TM4SF3, and knockdown of either STUB1 or CHIP had no effect (Fig. 1A). The importance of MDM2 E3 ligase activity was assessed using the HMDM2 E3 Ligase Inhibitor II HLI373 (MDM2i) (Kitagaki *et al.* 2008), which increased the levels of both AR and TM4SF3 proteins, similar to the effect of siRNA knockdown (Fig. 1B). Collectively, these data strongly suggest that MDM2 E3 ligase activity diminishes the protein levels of both AR and TM4SF3 in prostate cancer cells.

Since the TM4SF3 interaction with AR leads to mutual stabilization of both proteins (Bhansali *et al.* 2016, Khatiwada *et al.* 2023), it is possible that MDM2 is indirectly affecting the TM4SF3 protein via its direct effect on AR. To address this, we compared the effect of MDM2i on TM4SF3 in LNCaP cells to PC-3 cells, which do not express AR (Otsuka *et al.* 2013). Importantly, MDM2i had the same positive effect on TM4SF3 protein levels in PC-3 cells as in LNCaP cells (Fig. 1C), indicating that AR is not required for the effect and is consistent with the proposition that MDM2 is directly acting on TM4SF3.

We previously reported that androgen or MG132 promotes the stability of both AR and TM4SF3 (Bhansali *et al.* 2016). As expected, MDM2i had a similar positive effect on AR and TM4SF3 protein levels as did R1881 or MG132 (Fig. 1D). Interestingly, while AR and TM4SF3 protein levels were elevated upon MDM2i treatment in the absence of androgen, MDM2i had no effect in the presence of androgen (Supplementary Fig. 3A). Repeating this experiment with cycloheximide clearly showed that MDM2i stabilized both AR and TM4SF3 proteins, as did androgen treatment (Supplementary Fig. 3B). These data suggest that MDM2 may be involved in the ubiquitination of AR and TM4SF3, which we have previously shown to be disrupted by hormone treatment (Khatiwada *et al.* 2023). To examine the possibility that MDM2 affects ubiquitination, we monitored the ubiquitination of AR and TM4SF3, both of which were inhibited by MDM2i (Fig. 1F), strongly suggesting that MDM2 is inducing the ubiquitination of these two proteins.

### MDM2 regulates the protein stability of AR-V7

**Figure 2:**
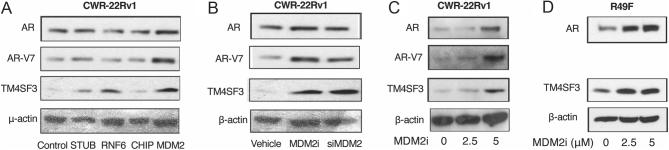
MDM2 regulates AR-V7 protein stability. CWR-22Rv1 cells grown in 2% DCC for 48 h were (A) transfected with siRNA, (B) treated with vehicle (DMSO), 5 μM MDM2i, or transfected with siMDM2, or (C) treated with 0 (vehicle), 2.5, or 5 μM MDM2i. (D) R49F cells grown in 2% DCC for 48 h were treated with 0 (vehicle), 2.5, or 5 μM MDM2i. After 48 h of transfection or treatment, cells were lysed with M-PER, and Western blotting was performed to detect AR, AR-V7, and TM4SF3 as indicated. In all Western blots, β-actin was used as a loading control.

Since a specific E3 ligase has not been identified for AR-V7, we studied the four known E3 ligases acting on AR in CWR-22Rv1 cells, which express both AR and AR-V7 (Djusberg *et al.* 2017). Depletion by siRNA of only MDM2 resulted in elevated protein levels of AR-V7, as well as AR and TM4SF3, in CWR-22Rv1 cells (Fig. 2A), mimicking the findings in LNCaP cells (Fig. 1A). MDM2 inhibition was equally effective as siRNA knockdown in elevating levels of AR-V7, as well as AR and TM4SF3, in CWR-22Rv1 cells (Fig. 2B). We further determined that the MDM2i enhancement effect is dose-dependent, as AR, AR-V7, and TM4SF3 levels increased with increasing concentrations of MDM2i (Fig. 2C). The same dose-dependent effect of MDM2i on AR and TM4SF3 was also observed in R49F cells (Fig. 2D), which, together with the CWR-22Rv1 cell data, clearly demonstrates that MDM2 regulates the levels of TM4SF3 and AR proteins in enzalutamide-resistant cells.

### MDM2 inhibition promotes the growth of prostate cancer cells in androgen-free conditions

**Figure 3:**
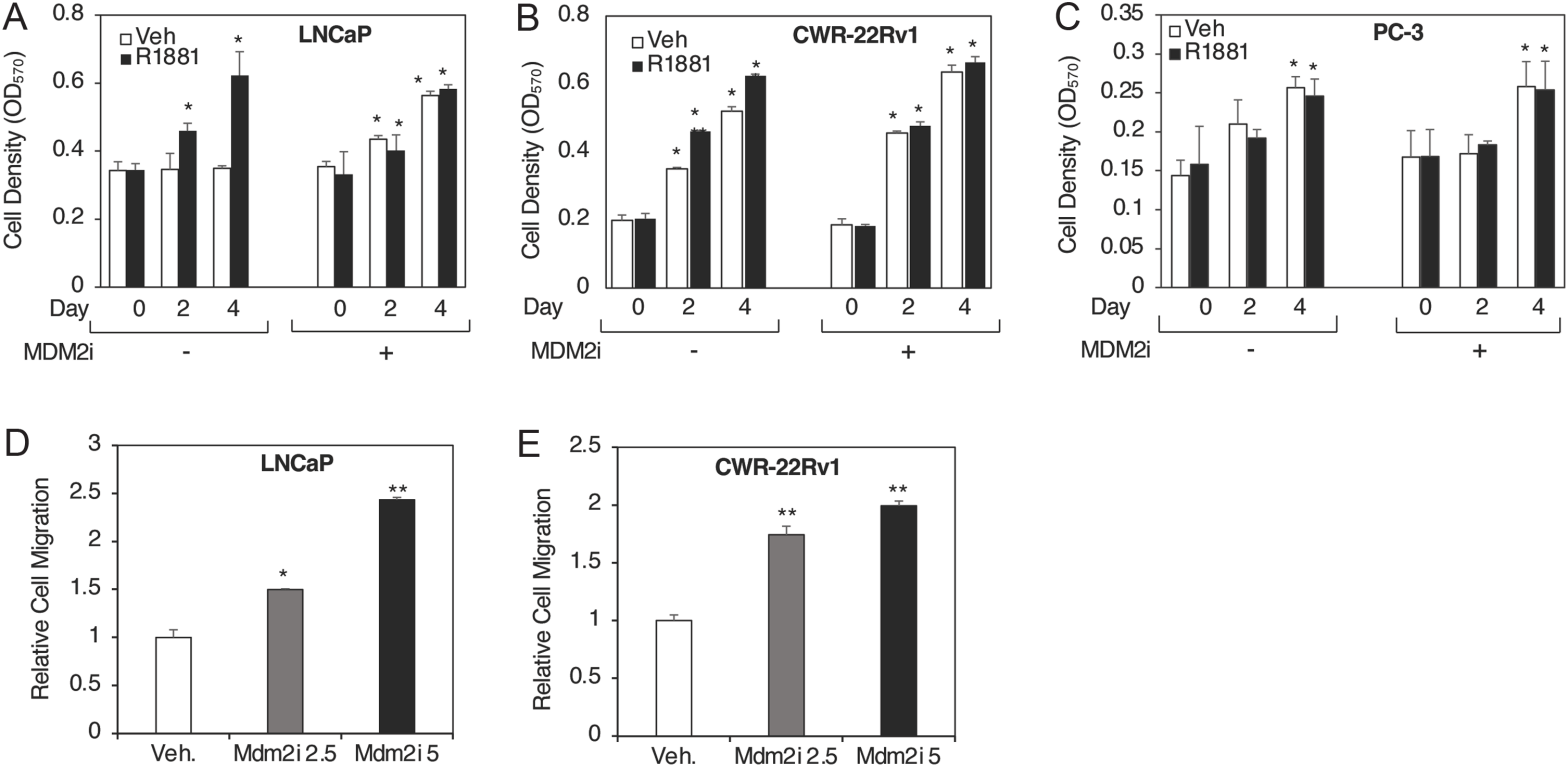
MDM2 inhibition promotes the growth of prostate cancer cells in androgen-free conditions. (A) LNCaP, (B) CWR-22Rv1, and (C) PC-3 cells grown in 2% DCC for 48 h were treated with vehicle (ethanol) or 10 nM R1881, and either DMSO (−) or 5 μM MDM2i (+). After 0, 2, or 4 days, cell proliferation was measured using an MTT proliferation assay. (D) LNCaP and (E) CWR-22Rv1 cells grown in 2% DCC for 48 h were treated with 0, 2.5, and 5 μM MDM2i for 48 h and monitored for migration, represented in bar graph after normalizing for proliferation. Bar graphs represent averages of three independent experiments plus s.d. A Student’s *t*-test was performed to show statistical significance ( *P* < 0.05), as indicated by the asterisks, (A, B, C) in the number of cells on day 2 or day 4 as compared to day 0 or (D, E) in cell migration with different concentrations of MDM2i as compared to vehicle.

AR signaling is critical for the viability of AR-expressing prostate cancer cells (Huang & Tindall 2002, Dehm & Tindall 2006). Since MDM2 inhibition increased the AR and AR-V7 protein levels, MDM2 inhibition is expected to affect the growth of prostate cancer cells. As expected, LNCaP cells did not grow in the absence of androgen but grew well in its presence (Fig. 3A). Interestingly, treatment with MDM2i promoted these cells to grow in the absence of hormone, and this growth was as good as the growth in the presence of hormone (Fig. 3A), even after 8 days of cell growth (Supplementary Fig. 4). In contrast, the growth of CWR-22Rv1 cells is largely androgen-independent, and MDM2i increased weakly but significantly the growth of these cells only in the absence of androgen (Fig. 3B). Not surprisingly, MDM2i treatment did not affect the proliferation of LNCaP (Supplementary Fig. 5A) and CWR-22Rv1 (Supplementary Fig. 5B) cells grown in full serum. AR-negative PC-3 cells were not significantly affected by MDM2i (Fig. 3C and Supplementary Fig. 5C).

Since AR is involved in prostate cancer cell migration (Guo *et al.* 2018), we were interested to determine if MDM2 inhibition would affect these processes. Treatment with MDM2i significantly increased and dose-dependently the migration of LNCaP (Fig. 3D) and CWR-22Rv1 cells (Fig. 3E). The results collectively show that MDM2 inhibition markedly enhances the migration of prostate cancer cells, which are likely mediated by the positive effect of MDM2i on AR, AR-V7, and TM4SF3 proteins.

### Inhibition of MDM2 increases the expression AR and AR-V7 target genes

**Figure 4:**
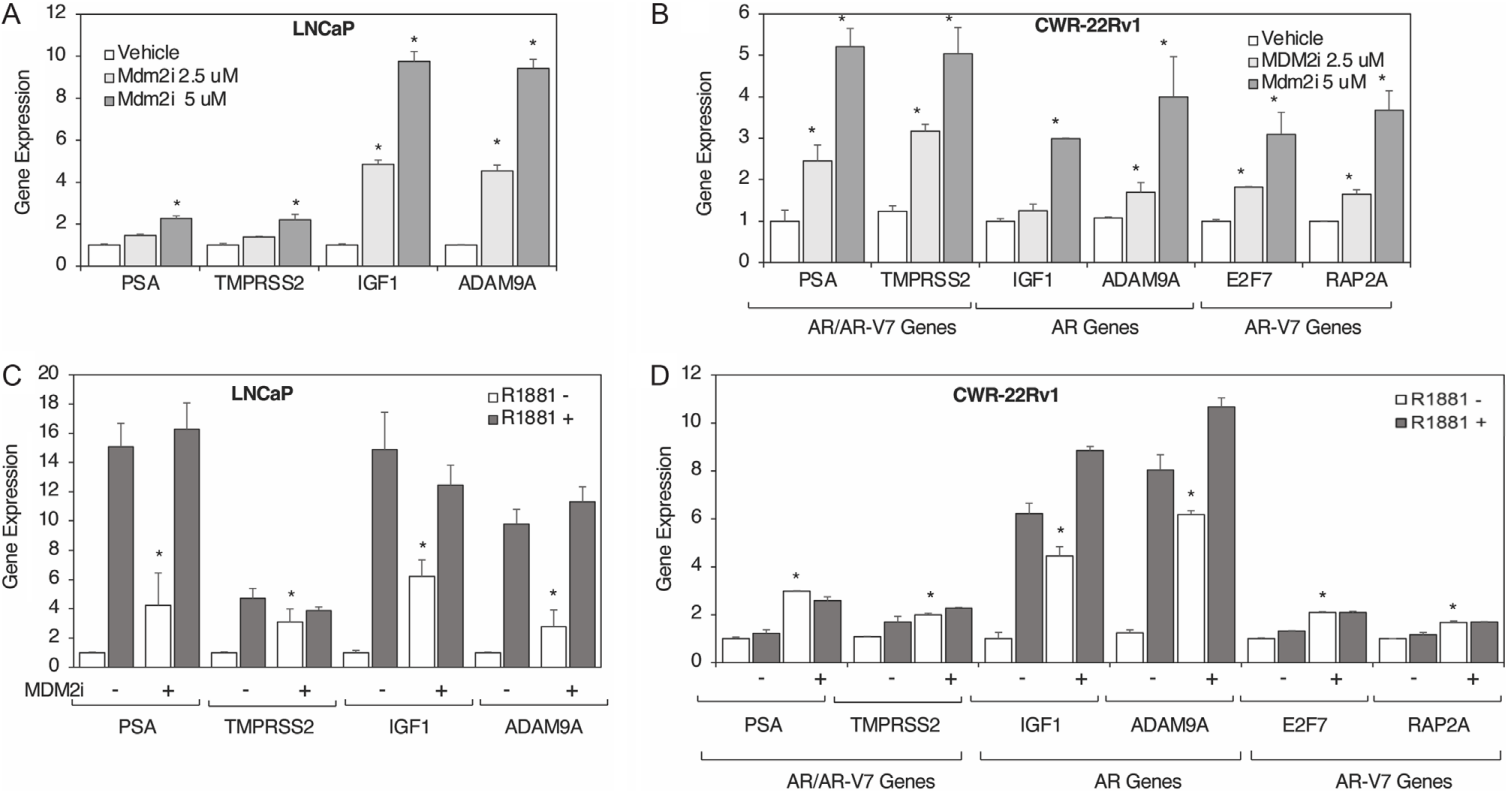
MDM2 inhibition increases the expression of AR and AR-V7 target genes in androgen-free conditions. (A, C) LNCaP and (B, D) CWR-22Rv1 cells grown in 2% DCC for 48 h were treated with (A, B) 0 (vehicle), 2.5, or 5 μM MDM2i or (C, D) ethanol (−) or R1881 (+) and DMSO (−) or 5 μM MDM2i (+) for 48 h before mRNA extraction and qRT-PCR to measure the expression of AR and AR-V7 target genes (as indicated in the text). Bar graphs represent averages of three independent experiments plus s.d. A Student’s *t*-test was performed to show statistical significance (*P* < 0.05), as indicated by the asterisks, in gene expression in the presence of MDM2i (+) as compared to vehicle (−).

Inhibition of MDM2 E3 ligase activity markedly increased AR and AR-V7 protein levels, as well as TM4SF3, which posed the important question of whether these increases would result in increased expression of AR and AR-V7 target genes. We first checked the effect of MDM2 inhibition on gene expression in the absence of androgen (cells grown in 2% DCC-FBS). Interestingly, MDM2i treatment increased, in a dose-dependent manner, the expression of PSA, TMPRSS2, IGF1, and ADAM9A in LNCaP cells (Fig. 4A). The increased expression induced by MDM2i was two-fold in PSA and TMPRSS2 and eight-fold in IGF1 and ADAM9A (Fig. 4A). Similar enhancing effects of MDM2i were observed in CWR-22Rv1 cells on the AR-V7 target genes E2F7 and RAP2A, in addition to AR or AR/AR-V7 target genes (Fig. 4B). AR, AR-V7, and TM4SF3 gene expression were not affected by the MDM2i treatment in LNCaP (Supplementary Fig. 6A) and CWR-22Rv1 (Supplementary Fig. 6B) cells grown in 2% DCC-FBS.

Since our earlier data (Fig. 1D) showed that MDM2 inhibition had the same enhancing effect on AR and TM4SF3 protein levels as does androgen, we were interested to evaluate the effect of MDM2i on gene expression in the absence or presence of androgen. In LNCaP cells, MDM2i markedly increased the expression of PSA, TMPRSS2, IGF1, and ADAM9A in the absence of androgen but had little effect in its presence (Fig. 4C). Similar but distinct results were obtained with CWR-22Rv1 cells, with MDM2i enhancing the expression of both AR and AR-V7 target genes in the absence and, to a lesser yet significant extent, in the absence of hormone (Fig. 4D). Interestingly, in CWR-22Rv1 cells, MDM2i had the stronger effects on AR target genes than on AR-V7 target genes (Fig. 4D). These results clearly show that MDM2 regulates the expression of both AR and AR-V7 target genes.

## Discussion

Androgen binding to AR leads to the disassociation of AR from heat shock proteins and AR dimerization and nuclear localization, where the homodimer binds to promoter and enhancer regions of androgen-regulated genes (Pratt & Toft 1997, Georget *et al.* 2002). Transcriptional regulation of such AR-regulated genes is essential for the progression of prostate cancer, and studies have shown that proper AR turnover is critical for maintaining regulated transcription of such genes (Huang & Tindall 2002, McEwan 2004). Polyubiquitination of AR by specific E3 ligases provides the recognition signal for the 26S proteasome, leading to AR protein degradation (Hershko & Ciechanover 1998). Several E3 ligases implicated in AR polyubiquitination have already been identified (Li *et al.* 2014). MDM2 was the first E3 ligase found to associated with AR, and MDM2-associated polyubiquitination regulates AR by promoting its proteasomal degradation (Giridhar *et al.* 2019). RNF6, which was identified as an AR-associated E3 ligase by proteomic screening (Kitagaki *et al.* 2008), and Siah2 are known to induce AR ubiquitination and promote AR transcriptional activity (Otsuka *et al.* 2013). Similarly, it has been reported that Aurora kinase promotes AR ubiquitination and degradation through CHIP (Djusberg *et al.* 2017).

In contrast to AR, TM4SF3 protein stability is largely unexplored. Our lab has published that the proteasomal inhibitor MG132 blocked TM4SF3 degradation (Bhansali *et al.* 2016), suggesting for the first time that TM4SF3 is a target of ubiquitination. More recently, we have demonstrated that TM4SF3 is, in fact, polyubiquitinated (Khatiwada *et al.* 2023). We have also reported that TM4SF3 directly interacts with AR, and the complex is protected from proteasomal degradation by inhibiting ubiquitination of both proteins (Khatiwada *et al.* 2023). These data led us to hypothesize that there may be a common E3 ligase that acts on both AR and TM4SF3, which is inhibited by the androgen-induced AR/TM4SF3 interaction. In our search for a common E3 ligase, we focused on those four ligases that have previously been shown to act on AR, and siRNA depletion experiments clearly showed that only MDM knockdown resulted in increased levels of both AR and TM4SF3 proteins in prostate cancer cells. The E3 ligase activity of MDM2 has many well-known targets (reviewed in Michael & Oren 2003, Li *et al.* 2014), including p53 and AR, and now we add TM4SF3 to the list.

MDM2 promotion of AR polyubiquitination has been previously reported (Giridhar *et al.* 2019). In this study, we verify that earlier finding by using either siRNA depletion or an MDM2i, both of which led to enhanced AR protein levels in prostate cancer cells. More importantly, our data here show that the MDM2 inhibitor had a similar effect on AR protein as androgen did, strongly suggesting that hormone stabilizes AR by blocking its MDM2-induced ubiquitination. Indeed, we determined that AR ubiquitination was markedly reduced following treatment with MDM2i. The results suggest that MDM2 is an E3 ligase inducing AR polyubiquitination and protein degradation in prostate cancer cells.

Our data in this study suggest that TM4SF3 is also a target of MDM2 E3 ligase activity. These data include siRNA knockdown and treatment with MDM2i, both of which led to elevated levels of endogenous TM4SF3 in prostate cancer cells. While these findings suggest that MDM2 is acting directly on TM4SF3, it is also possible that MDM2 is acting indirectly on TM4SF3 through its direct effect on AR, whose elevated levels can lead to increased TM4SF3 protein through its interaction with TM4SF3 (Bhansali *et al.* 2016). This possibility was examined by repeating the above experiment in PC-3 cells, which do not express endogenous AR (Otsuka *et al.* 2013). Importantly, MDM2 inhibition by siRNA or MDM2i treatment also resulted in elevated TM4SF3 protein in PC-3 cells, demonstrating that the MDM2 effect on TM4SF3 is independent of AR. In support of this direct effect, we determined here that MDM2 affects the ubiquitination status of TM4SF3, and MDM2 interacts with TM4SF3 in prostate cancer cells.

As prostate cancer progresses into the lethal CRPC stage, androgen becomes non-essential for the activation of AR due to several mechanisms, including the expression of constitutively active splice variants of AR (Sharifi 2013). AR-V7 is the major splice variant of AR, which lacks the LBD and thus does not require androgen for its activation. AR-V7 lacks two (K845 and K847) of the three (K311, K845, and K847) lysine residues known to be ubiquitinated on AR (McClurg *et al.* 2017). While some studies have suggested proteasomal degradation of AR-V7 (Liu *et al.* 2018), the underlying mechanism for AR-V7 turnover is not fully understood (Moon *et al.* 2018). While STUB1 (Liu *et al.* 2018) and MDM2 (Li *et al.* 2015) have been reported to promote AR-V7 proteasomal degradation, an E3 ligase directly involved in AR-V7 polyubiquitination has not been identified (Liu *et al.* 2018). Consistent with that earlier study (Li *et al.* 2015), our data here show that AR-V7, like AR and TM4SF3, is under MDM2 regulation, since siRNA knockdown or MDM2i treatment of CWR-22Rv1 cells resulted in elevated levels of endogenous AR-V7. AR-V7 ubiquitination, which only occurs in the presence of co-expressed AR (Khatiwada *et al.* 2023), is influenced by MDM2, and AR-V7 was found to interact with MDM2. Since MDM2 has been previously shown to ubiquitinate through AR lysine 311 (McClurg *et al.* 2017), which is also found on AR-V7, then it is possible that MDM2 regulates AR-V7 protein through ubiquitination of K311. It is also important to mention that we have previously shown that TM4SF3 can interact with AR-V7 and undergo mutual stabilization (Khatiwada *et al.* 2023), as it does with AR. Thus, it is possible that the AR-V7/TM4SF3 complex also disrupts MDM2 activity on these two proteins.

AR and AR-V7 play a critical role in the viability of prostate cancer cells (Takayama & Inoue 2013). Because MDM2 inhibition increased protein stability of both AR and AR-V7, it was not surprising to learn that MDM2 inhibition caused increased proliferation and migration of LNCaP and CWR-22Rv1 cells. As expected, these effects were only observed when cells were grown without androgen, since MDM2 inhibition had no significant effect on AR, AR-V7, or TM4SF3 protein levels with androgen. PC-3 cells, which do not express AR, responded with elevated levels of TM4SF3 in response to MDM2 inhibition, but this had no significant effect on cell proliferation, clearly demonstrating that TM4SF3 without endogenous AR does not affect the viability of prostate cancer cells. The effects of AR and AR-V7 on prostate cancer cell viability and migration are mediated by their impact on gene expression (Lu *et al.* 2015). Thus, it was unsurprising that MDM2 affected gene expression in prostate cancer cells. Indeed, MDM2 inhibition led increased expression of the AR-regulated genes PSA, TMPRSS2, IGF1, and ADAM9A in LNCAP cells. Similarly, MDM2 inhibition increased in CWR-22Rv1 cells the expression of both AR and AR-V7 target genes, including the (i) AR- and AR-V7-regulated PSA and TMPRSS2, (ii) AR-regulated IGF1 and ADAM9A, and (iii) AR-V7-regulated E2F7 and RAP2A (Lu *et al.* 2015).

This study identifies MDM2 as a potential common E3 ligase for AR, AR-V7, and TM4SF3 in prostate cancer cells and suggests a novel mechanism for the mutual stabilization of TM4SF3 with AR or AR-V7 by disrupting the action of MDM2 on these proteins. Future work will focus on demonstrating the direct action of MDM2 on these three proteins through *in vitro* assays . We will also focus on identifying the ubiquitination sites of TM4SF3 and possible new ubiquitination sites on AR and AR-V7, and determining the type of polyubiquitination chain.

## Supplementary materials

Supplementary Figures

## Declaration of interest

There is no conflict of interest for all the coauthors.

## Funding

This work was supported by a grant from the U.S. Department of Defensehttp://dx.doi.org/10.13039/100000005 (W81XWH-17-1-0263).

## Author contribution statement

PK performed experiments and analyzed data; UR performed experiments and analyzed data; ZH performed experiments; and LS conceived the study and wrote the manuscript.
