# Supplementary material for: MDM2 regulates the stability of AR, AR-V7, and TM4SF3 proteins in prostate cancer
Source: Endocr Oncol. 2024 Feb 9;4(1):e230017. doi: 10.1530/EO-23-0017 (PMC10895308; doi:10.1530/EO-23-0017)
Supplement: Supplementary Figures [file supplementary_figures.pdf]

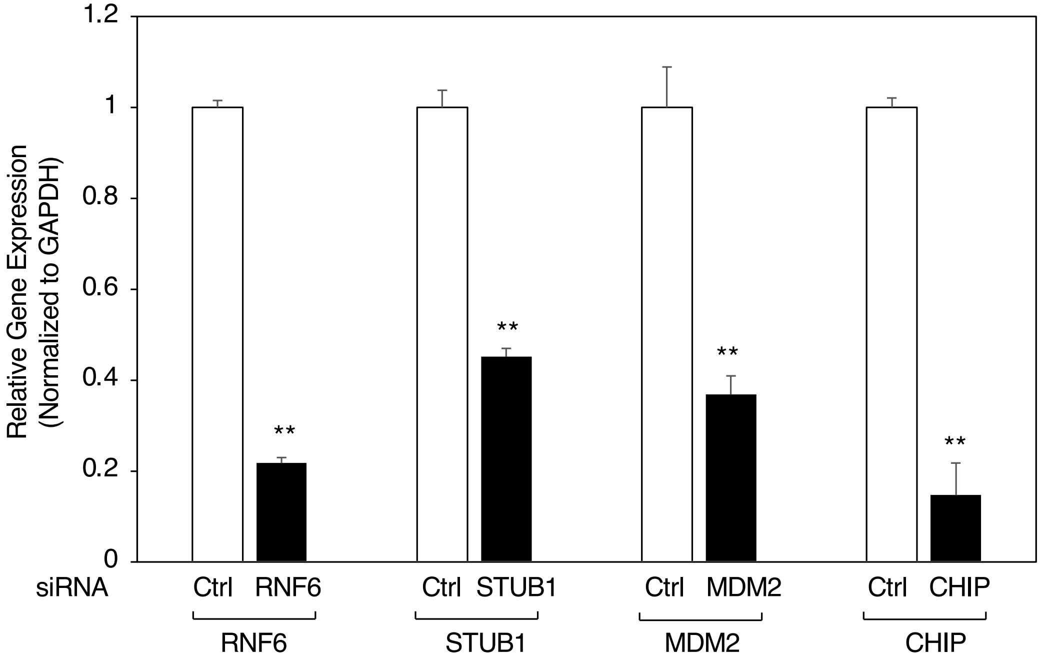

Fig. S1. Knockdown or Inhibition of E3 Ligases. LNCaP cells grown in 2% DCC for 48 hours were transfected with indicated control (Ctrl) siRNA or siRNAs targeting a specific E3 ligase, as indicated. After 48 hours, mRNA was extracted and gene expression of RNF6, STUB1, MDM2, or CHIP were measured by qRT-PCR. Bar graphs represent averages of 3 independent experiments plus standard deviation. The Student's T-test was performed to show statistical significance (**=$p \leq 0.01$) in siRNA-treated cells, as indicated by the asterisks.

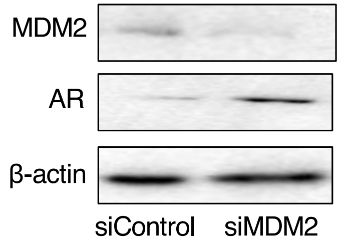

Fig. S2. siRNA knocks down MDM2 protein levels in prostate cancer cells. LNCaP cells were grown for 48 hours before transfection with indicated siRNA Control or siRNA against MDM2. After 48 hours of treatment, cells were lysed with M-PER®, and AR and TM4SF3 protein levels were measured by Western blotting. Note that β-actin was used as a loading control for the Western blots.

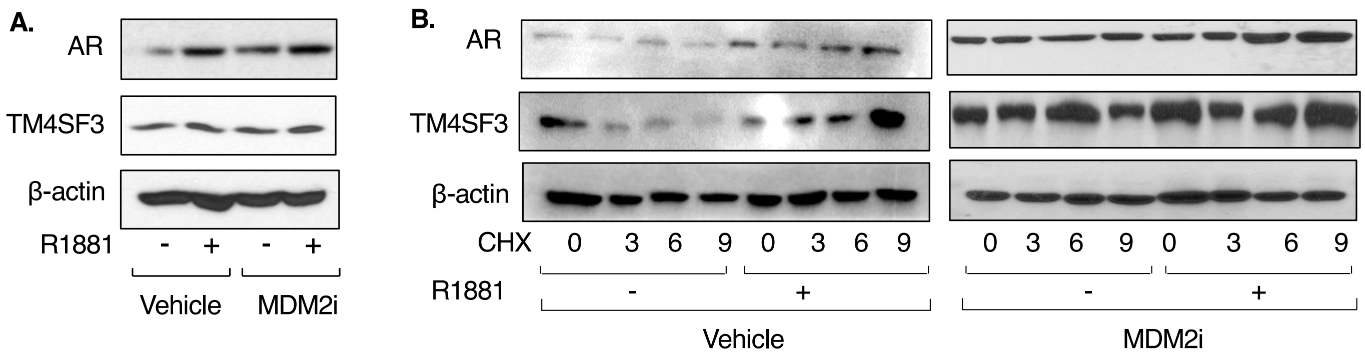

Fig. S3. MDM2 inhibition increases stabilizes both the AR and TM4SF3 protein levels in prostate cancer cells. LNCaP cells grown in 2% DCC for 48 hours were treated with ethanol (-) or 10 nM R1881 (+), and either DMSO or 5 μM MDM2i in the absence (**A**) or presence of 20 μM Cycloheximide (CHX) (**B**). After 48 hours of treatment, cells were lysed with M-PER®, and AR and TM4SF3 protein levels were measured by Western blotting. Note that β-actin was used as a loading control for the Western blots.

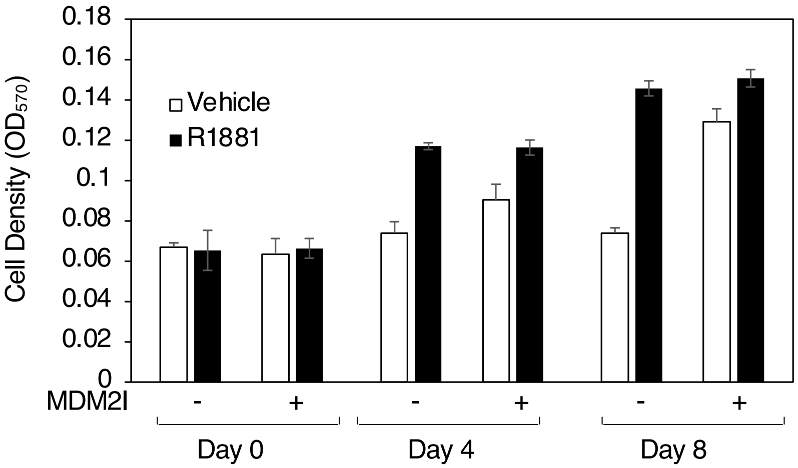

Fig. S4. MDM2 Inhibition has a similar positive effect as androgen treatment on the growth of prostate cancer cells. LNCaP grown in full serum were treated with 0, 2.5, and 5 μM MDM2i. After 0, 4, or 8 days of treatment cell density was measured by MTT proliferation assay. Bar graphs represent averages of 3 independent experiments plus standard deviations. The Student's T-test was performed to show no statistical significance in MDM2i-treated cells.

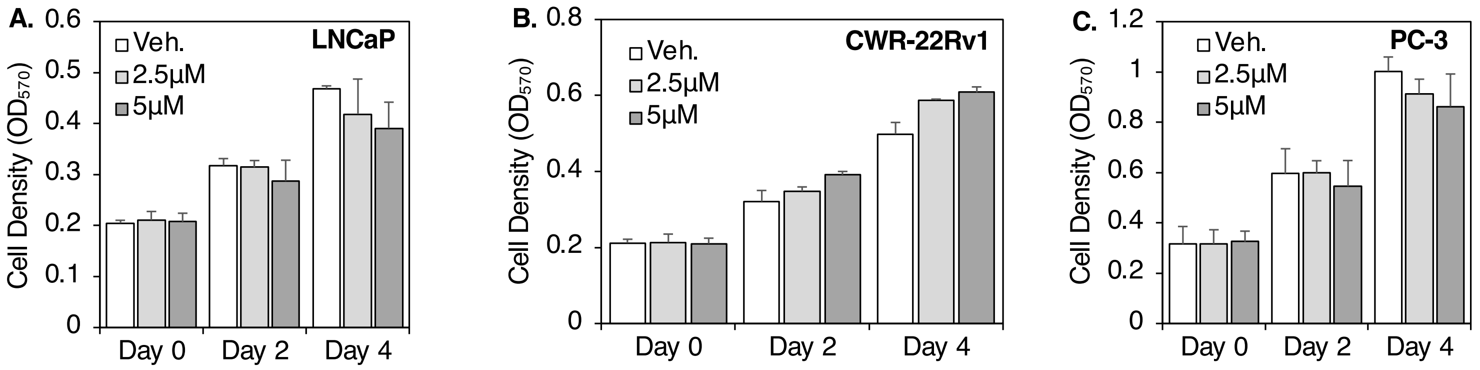

Fig. S5. MDM2 Inhibition Does Not Affect Cells Grown in Full Serum. LNCaP (**A**), CWR-22Rv1 (**B**), and PC-3 (**C**) cells grown in full serum were treated with 0, 2.5, and 5 μM MDM2i. After 0, 2, or 4 days of treatment cell density was measured by MTT-proliferation assay. Bar graphs represent averages of 3 independent experiments plus standard deviations. The Student's T-test was performed to show no statistical significance in MDM2i-treated cells.

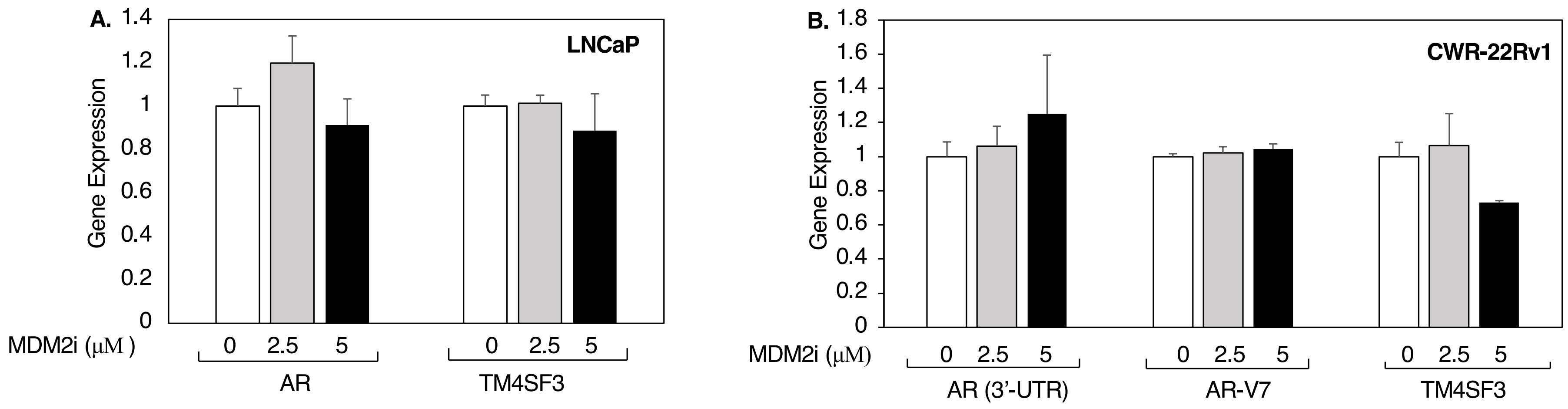

Fig. S6. MDM2i Treatment Does Not Affect AR/AR-V7, or TM4SF3 Gene Expression. LNCaP (**A**) and CWR-22Rv1 (**B**) cells grown in 2% DCC for 48 hours were treated with 0, 2.5, and 5 μM MDM2i for 48 hours before mRNA extraction following which AR, AR-V7 and TM4SF3 expression were measured as indicated by qRT-PCR. Bar graphs represent averages of 3 independent experiments plus standard deviation. The Student's T-test was performed to show there was no statistical significance in MDM2i-treated cells.
